# Spinal Postures and Mobility in Children with Achondroplasia vs. Age- and Sex-Matched Healthy Individuals: A Preliminary Report

**DOI:** 10.3390/jcm13072135

**Published:** 2024-04-07

**Authors:** Munkh-Erdene Bayartai, Hannu Luomajoki, Andrea Aliverti, Antonella LoMauro, Gabriella Tringali, Alessandro Sartorio

**Affiliations:** 1 Institute of Physiotherapy, School of Health Professions, Zurich University of Applied Sciences, 8400 Winterthur, Switzerland; luom@zhaw.ch; 2 Department of Physical and Occupational Therapy, School of Nursing, Mongolian National University of Medical Sciences, Ulaanbaatar 14210, Mongolia; 3 Department of Electronics, Information and Bioengineering, Politecnico di Milano, 20133 Milan, Italy; andrea.aliverti@polimi.it (A.A.); antonella.lomauro@polimi.it (A.L.); 4 Istituto Auxologico Italiano, Istituto di Ricovero e Cura a Carattere Scientifico (IRCCS), Experimental Laboratory for Auxo-Endocrinological Research, 28824 Piancavallo-Verbania, Italy; g.tringali@auxologico.it (G.T.); sartorio@auxologico.it (A.S.)

**Keywords:** achondroplasia, children, spinal mobility, spinal posture

## Abstract

**Background:** Achondroplasia is a rare genetic disease, yet the most common form of dwarfism, characterized by limb shortening and disproportionate short stature along with musculoskeletal changes, such as postural deviations. Although postural changes in the spine in children with achondroplasia have been well investigated, little is known about the association of achondroplasia with spinal movements/mobility. **Methods:** This preliminary study aims to explore the association of achondroplasia with spinal mobility in children with achondroplasia compared to age- and sex-matched healthy individuals. Spinal posture and mobility were assessed using a radiation-free back scan, the Idiag M360 (Idiag, Fehraltorf, Switzerland). Between-group differences were determined using a two-way analysis of variance. **Results:** Children with achondroplasia had smaller thoracic lateral flexion [difference between groups (Δ) = 20.4°, 95% CI 0.1°–40.6°, *p* = 0.04], lumbar flexion (Δ = 17.4°, 95% CI 5.5°–29.4°, *p* = 0.006), lumbar extension (Δ = 14.2°, 95% CI 5.7°–22.8°, *p* = 0.002) and lumbar lateral flexion (Δ = 19.6°, 95% CI 10.7°–28.4°, *p* < 0.001) than age- and sex-matched healthy individuals, except for thoracic extension (Δ = 16.5°, 95% CI 4.4°–28.7°, *p* = 0.009) which was greater in children with achondroplasia. No differences were observed in global spinal postures between the two groups. **Conclusions:** Spinal mobility appears to be more influenced by achondroplasia than global spinal postures in childhood. These results also highlight the importance of considering the musculoskeletal assessment of segmental spinal postures and rehabilitative interventions aimed at promoting spinal flexibility in children with achondroplasia.

## 1. Introduction

Achondroplasia is a rare genetic disease, yet the most common form of dwarfism, characterized by limb shortening and disproportionate short stature along with changes and impairments in musculoskeletal, neurological as well as cardiovascular systems leading to various health issues [1,2,3]. Achondroplasia is caused by autosomal dominant mutations in the fibroblast growth factor receptor 3 (FGFR3 gene), hindering the normal process of endochondral bone growth [1,2]. The prevalence of achondroplasia is estimated to be about 1 in 25,000 births, with more than 250,000 individuals globally [1,4,5,6].

Physical characteristics of individuals with achondroplasia include disproportionate shortening of the upper limbs [7], disproportional limb-to-trunk ratio as well as impairments/changes in multiple body systems, varying from birth to adulthood [3]. For example, common characteristics presented by infants with achondroplasia include gross motor delay, hypotonia with weakness, foramen magnum stenosis, otitis media, hearing deficit, sleep-disordered breathing, upper airway obstruction and alterations in spinal postures, such as kyphosis [8]. A review concerning the lifetime impact of achondroplasia across the lifespan found that common comorbidities associated with achondroplasia in infancy and childhood are gross motor delay, hypotonia with weakness, ventriculomegaly and otitis media, whilst impaired physical function, impaired social functioning, pain, obesity, lower quality of life, musculoskeletal changes such as hip flexion contracture as well as genu varum frequently occur in childhood, adolescence, and adulthood [8]. A recent research study involving 42 parents/caregivers of individuals with achondroplasia and 19 healthcare practitioners responsible for the care and management of achondroplasia in Italy indicated that otitis, foramen magnum stenosis, hearing deficit, and sleep apnoea were reported by parents/caregivers as the most remarkable complications associated with achondroplasia although the occurrence of these complications varies across the lifespan [9]. Parents/caregivers of children with achondroplasia reported that children with achondroplasia also experience difficulty in performing specific functional skills, such as reaching the top of their head as well as the middle of their back due to disproportional shortening of the upper limbs, which interferes with functional performance and self-care skills, such as hair brushing, dressing, bathing, and toileting [3,7,10]. Therefore, children diagnosed with achondroplasia necessitate increased physical support for their day-to-day activities, indicating a greater level of caring burden/responsibility for families [10]. According to the parents or caregivers of children with achondroplasia, the most notable influence of complications associated with achondroplasia was experienced by their children during the ages of 2 to 5 years [9], whilst children diagnosed with achondroplasia show a considerable enhancement in their overall functioning and various specific functional skill areas from the age of 3 to 5 years [10]. Increased accessibility to physiotherapy, occupational therapy, and speech therapy services have the potential to support greater independence in children with achondroplasia [10].

The spine is also commonly affected by achondroplasia, and spinal manifestations of achondroplasia include alterations in spinal postures and mobility. Lumbosacral lordosis, which is contributed by restricted hip flexion, potentially leading to back pain and muscle fatigue, is often experienced by individuals with achondroplasia [3,11]. Another common postural alteration in children with achondroplasia is thoracolumbar kyphosis, commonly present in newborns and infants with achondroplasia within the first 6 months of age, which However, significantly improves or vanishes in most children when they become able to stand and walk [12]. These postural changes potentially lead to low back pain, which is often associated with restricted spinal mobility, particularly reduced lumbar motion [13]. Achondroplasia appears to be associated with reduced spinal mobility. For example, a case report presented that a 7-year-old girl with achondroplasia had ossification of the anterior and posterior spinal ligaments, revealed by magnetic resonance imaging, and reduced spinal mobility, being linked to mild knee and back pain [14]. In addition to alterations in spinal postures, individuals with achondroplasia commonly experience obesity, muscle hypotonia, and hip flexion contractures that can restrict spinal mobility. Although postural changes in children with achondroplasia have been well investigated, studies specifically exploring the association of achondroplasia with spinal movements/mobility are lacking. Therefore, the present study aims to explore the association of achondroplasia with spinal mobility in children with achondroplasia compared to age- and sex-matched healthy individuals.

## 2. Materials and Methods

The present study employed a cross-sectional design to investigate the association of achondroplasia with spinal posture and mobility and followed the Strengthening the Reporting of Observational Studies in Epidemiology (STROBE) guidelines.

### 2.1. Participants

Children with achondroplasia were recruited during the annual meeting of the Association for the Information and Study of Achondroplasia (AISAC), held on 13–14 May 2023, in Rimini, Italy. Normal-weight participants were recruited from elementary schools and sports clubs in the Canton of Zurich, Switzerland. Participants were excluded if they had past and present musculoskeletal and neurological disorders/conditions influencing the spine and spinal motion characteristics, including spinal scoliosis and limb length discrepancy. The study followed the Helsinki Declaration of 1975, as revised in 2008, and was approved by the Ethics Committee of Politecnico of Milan, Italy (research code no. 13/2023) for the subjects with achondroplasia and by the Ethics Committee of Zurich (BASECno. 2018-00979) for healthy controls. The research procedure was explained to each participant and written informed consent was obtained by participants and their parents when it was appropriate.

### 2.2. Measurements

The Idiag M360 scan tool (Idiag, Fehraltorf, Switzerland) was used to evaluate spinal posture and the mobility of the spine as well as hip movements [15]. Spinal postures were determined from an upright standing position, while the assessment of spinal mobility was conducted during dynamic activities such as flexion, extension, and lateral bending. The Idiag M360, a radiation-free and non-invasive device, is a reliable tool that assesses spinal posture and mobility. The device records the angles of each vertebral joint and sacral slope through computer-assisted analysis while two rolling wheels embedded in the device follow the vertebral spinous processes from the seventh cervical vertebra to the third sacral vertebra during the measurement. The positions and movements of every individual segment in the sagittal and frontal planes are determined during the recording, and the data are sampled at a frequency of 150 Hz [15,16]. These measurements are then converted using an analogue–digital converter and transferred to a personal computer for analysis.

The spinal characteristics (including the mobility of the spine and hips) were assessed in both the longitudinal and coronal planes. Participants were guided to perform forward bending (flexion), spinal extension, and bending to both sides while standing upright. This procedure followed the protocol provided by previous research works for evaluating spinal posture and movement [15,17,18]. The Idiag staff trained both evaluators using educational videos to assess spinal posture and mobility both in healthy children and in subjects with achondroplasia.

The validity and reliability of the device have previously been established for measuring spinal posture and mobility in a wide variety of participants, including normal-weight, overweight individuals as well as people with obesity [15,16,19,20]. Validity research on spinal curvature measurements using X-ray examinations demonstrated a strong correlation between radiography measurements and those obtained with a spinal mouse [15,19]. Spinal ranges of motion previously reported in the literature align closely with the values estimated by the Idiag M360 [16]. Moreover, an earlier comparative research work on the device’s validity and reliability for assessing lumbar flexion found that both segmental and global lumbar movements measured by radiography were aligned closely with the values estimated by the device [20]. The device also demonstrated fair-to-good reliability when assessing spinal curvature and movement patterns in non-obese individuals [16]. In this research, the average standard error of measurement was around 2°, while intraclass correlation coefficients for the inter-examiner reliability ranged from 0.63 to 0.93.

### 2.3. Data Processing

Each range of motion value for the spinal parameters was determined in the frontal and sagittal planes by the difference between the range of motion measurements at the standing position and the end of motion ranges for each spinal segment. Overall, global ranges of the motion of the spine, including lumbar and thoracic movements, were calculated by summing 5 values of segmental ranges of motion for the lumbar spine and 12 for the thoracic spine. The tilt of the sacrum occurred at the end of lumbar flexion, and extension from the standing position was estimated as hip flexion and extension, respectively.

### 2.4. Statistical Analysis

Descriptive and inferential statistical analyses were performed using R software (version 4.3.2) [21]. R is an open-source, free statistical programming tool used for data manipulation, computation, analysis, and visualization and was found to be reliable for parameter estimation [22,23]. Descriptive statistics provided mean values and standard deviations (SD) as well as the number of individuals in each group and percentage N (%) for factors such as participants’ age, gender, spinal posture, movement, hip motion, and the lumbar-to-hip ratio. Data normality was checked by using the Shapiro–Wilk test. To compare demographic and anthropometric characteristics between the group of subjects with achondroplasia and the group of healthy subjects, the independent samples t-test was used for data with a normal distribution, Wilcoxon’s rank sum test for non-normally distributed data, and Pearson’s chi-square test for categorical variables. Multiple regression models, accounting for age and gender, were used to investigate the relationship between achondroplastic traits and spinal movements. A two-way analysis of variance (ANOVA), adjusted for age and gender, was used to evaluate significant differences in spinal posture and mobility between the two groups. Pairwise post hoc tests were performed using the R package “emmeans” (v 1.6.3) to compare the groups following the ANOVA analyses [24]. A *p*-value below 0.05 was considered statistically significant. Post hoc power analysis using the G*Power 3.1 software [25] showed that, given the sample size (n = 26) and the observed effect size (f = 0.52) in the present study, power was 72%.

## 3. Results

**Figure 1:**
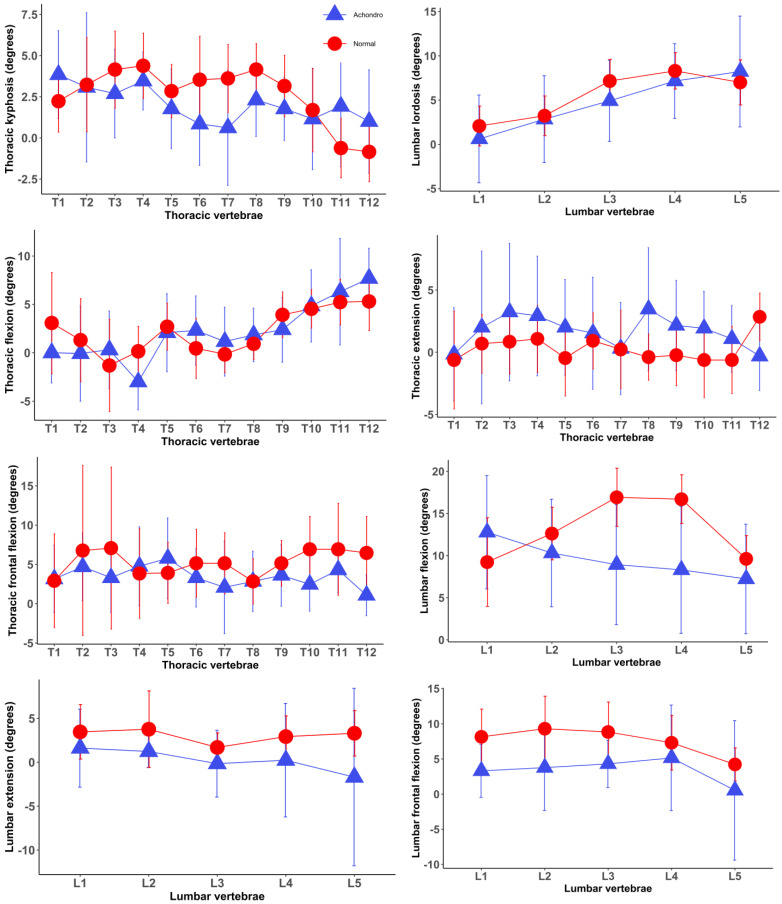
Posture and motion/kinematics of each individual spinal segment in the group of children with achondroplasia and the group of healthy children (mean and standard deviations). Positive and negative values indicate kyphosis/flexion and lordosis/extension, respectively. Normal—healthy children, Achondro—children with achondroplasia.

A total of 13 children with achondroplasia and 13 healthy children participated in the study. Participants’ characteristics are provided in Table 1. No differences in the mean age, BMI, sex ratio, and spinal length were observed between healthy children and children with achondroplasia, whereas the mean weight and height were higher in healthy children. Segmental posture and movements of the spine among healthy children compared to those with achondroplasia are displayed in Figure 1. Differences between the two groups were commonly noticed in the segmental movements of thoracic and lumbar vertebrae. For example, healthy children demonstrated greater segmental movements in lumbar vertebrae but less thoracic extension compared to those with achondroplasia.

### Associations of Achondroplastic Traits with Spinal Posture and Movements as well as Hip Motion

Statistically significant differences in some segmental spinal postures were observed between children with achondroplasia and healthy children, whereas no differences in global spinal postures were found between the two groups. Individuals with achondroplasia had greater segmental kyphotic angles of the spine in thoracic vertebrae T11/12 and T12/L1 but smaller segmental kyphotic angles in thoracic vertebrae T6/7 and T7/8 compared to those of healthy children. No differences in segmental angles of the remaining thoracic and lumbar vertebrae were observed between the two groups.

Statistically significant differences were observed in spinal movements as well as hip mobility between the healthy and achondroplastic groups, while spinal postures were not different between the two groups (Table 2). Healthy children had greater lumbar flexion, extension, and lateral flexion as well as thoracic lateral flexion than those with achondroplasia, while thoracic extension was greater in the group of subjects with achondroplasia. No statistically significant differences were observed in thoracic flexion between the two groups. The hip extension was also smaller in the group of subjects with achondroplasia than in the group of healthy subjects, while hip flexion was comparable in the two groups. In the post hoc test, the greatest differences observed among spinal movements were thoracic and lumbar lateral flexion, followed by lumbar flexion and extension (Table 2). Ranges of movement in thoracic and lumbar lateral flexion were 20.4° and 19.6°, respectively, being greater in healthy children, whereas thoracic extension was 16.50 larger in children with achondroplasia. No statistically significant differences were observed between the two groups in lumbar to hip ratio.

## 4. Discussion

The purpose of the present study was to explore the relationship between achondroplasia and spinal posture as well as motion/kinematics in children with achondroplasia in comparison with age and sex-matched healthy individuals, with the key finding being that achondroplasia was associated with alterations in spinal and hip mobility as well as segmental postures of the spine. Children with achondroplasia had smaller spinal and hip mobility than healthy individuals, except for thoracic extension, which was greater in children with achondroplasia. These findings suggest that achondroplasia appears to influence spinal mobility more than global spinal postures, and that it is important to take account of spinal mobility as well as segmental spinal postures in the musculoskeletal assessment of achondroplasia in childhood.

Achondroplasia was associated with increased thoracic extension and decreased thoracic lateral flexion, but no significant association was observed with thoracic flexion. Reductions in thoracic flexibility/movements are associated with low back pain, commonly occurring in individuals with achondroplasia [26]. A previous clinical trial of adults with chronic low back pain found that interventions designed to improve thoracic flexibility, such as mobilization or manipulation positively influenced lumbar mobility, which resulted in an improvement in disability and mental state in people with low back pain [27]. Another clinical trial involving 70 volleyball players, aged between 15 and 17, from high schools showed that participants assigned to the intervention group where they engaged in preventative exercises designed to improve thoracic dynamic mobility and stabilization had a significantly lower incidence of low back pain in the follow-up period of 4 weeks [28]. Therefore, reduced thoracic flexibility observed in the present study could be a contributing factor to low back pain in individuals with achondroplasia. In addition, thoracic mobility is essential for performing various tasks of daily activities and engaging in sports [29]. Therefore, restrictions/impairments in thoracic mobility could be a contributing factor to the decline in the ability to self-care in individuals with achondroplasia [3,7,10]. The present study found that participants with achondroplasia had greater thoracic extension, which may be explained by the compensation to thoracolumbar kyphosis occurring commonly in infants with achondroplasia, even though this postural deviation significantly improves or vanishes in most children when they become able to stand and walk [12]. However, studies investigating the association of achondroplasia with spinal movements to date are sparse, suggesting that further studies with larger sample sizes are needed to confirm the findings from the present study.

Lumbar flexion, extension, and lateral flexion were found to be smaller in children with achondroplasia than in healthy children. Studies specifically investigating achondroplasia in relation to lumbar mobility are also lacking to date. Changes or alterations in lumbar mobility are commonly associated with musculoskeletal conditions, particularly low back pain interfering with various tasks of daily activities where lumbar movements play an important role [13,30]. Low back pain is commonly experienced by individuals with achondroplasia [26]. Prospective studies have shown that restricted lumbar mobility, particularly in the frontal plane, amplifies the risk/chances of developing low back pain [13]. In the present study, the reductions in lumbar mobility in individuals with achondroplasia could potentially predispose them to low back pain. Overall, the findings suggested that achondroplasia appears to contribute to impairments in lumbar mobility in the sagittal and frontal plane, which may be taken into account when developing strategies designed to prevent a reduction in the lumbar mobility associated with achondroplasia and promote lumbar flexibility in this pediatric population. However, further longitudinal studies exploring the association of achondroplasia with low back pain, taking account of lumbar mobility, would help to better understand the factors predisposing individuals with achondroplasia to low back pain.

Achondroplasia was found to be associated with reduced hip extension, whereas no associations were observed with hip flexion as well as the lumbar-to-hip ratio. Previous literature available to date reported that children with achondroplasia demonstrate limited hip extension compared to healthy children [3], which was in line with the findings from the present study. Additionally, hip flexion contractures occur in children with achondroplasia, which is believed to be due to uncorrected lumbosacral lordosis [26]. This postural deviation in the hip joint could be a contributing factor to reducing hip extension in children with achondroplasia. Hip extension flexibility is also essential to efficiently perform important functional activities such as walking, climbing stairs, and hiking [31]. A cross-sectional study exploring gait kinematics using three-dimensional gait analysis indicated that children with achondroplasia were associated with reduced gait speed, step length, and increased cadence [32]. Reduced hip extension observed in the present study could potentially contribute to these alterations in gait. Impairment in hip extension flexibility is commonly associated with the ineffective performance of functional tasks due to impaired walking economy and musculoskeletal conditions such as low back pain [33,34,35]. Although hip extension was found to be smaller in children with achondroplasia, no statistically significant differences were identified in hip flexion and the lumbar-to-hip ratio, implying that achondroplasia may influence hip extension more than hip flexion and the lumbar-to-hip ratio.

Segmental spinal postures were different between children with achondroplasia and healthy children, whereas no differences between the two groups were observed in global spinal postures. Segmental kyphotic angles of the spine in individuals with achondroplasia were larger in thoracic vertebrae T11/12 and T12/L1, whilst healthy children had greater segmental kyphotic angles in thoracic vertebrae T6, T7 and T8, suggesting that the spinal postures of participants with achondroplasia in the present study demonstrated thoracolumbar kyphosis, one of the typical postural alterations associated with achondroplasia [12]. Although thoracolumbar kyphosis is one of the most common alterations in the spine of infants with achondroplasia, this improves as children with achondroplasia become able to start walking [3,12]. Global spinal postures/curvatures were not different between children with achondroplasia and healthy children in the present study, implying that participants with achondroplasia had improved to near-normal spinal curvatures, which resulted in comparable global spinal postures compared to those of healthy children. These findings also highlight the importance of taking into account the evaluation of segmental spinal postures in the management of spinal alterations in children with achondroplasia.

We acknowledge that the present study has several limitations. The current study employed a cross-sectional design, which cannot provide evidence on whether the nature of the association between achondroplasia and alterations in spinal mobility is causal. The device used in the current study is designed to measure spinal posture and movements in the sagittal and frontal planes. Therefore, we were not able to measure spinal movements (rotation) in the horizontal plane. The present study explores spinal posture and mobility only in children, thus hampering a generalization of the results to people outside this age range. The sample size was relatively small, mitigating statistical power, which could be improved by future studies with a larger sample size. However, the study was strengthened by providing detailed information about the characteristics of the spine such as the global and segmental posture of the spine and spinal mobility in children with achondroplasia (a rare genetic disorder) in comparison with healthy children, as research works (available to date) exploring the association of achondroplasia with spinal movements/mobility are lacking.

In conclusion, achondroplasia was associated with spinal and hip mobility alterations in children. Children with achondroplasia had smaller spinal and hip mobility than healthy individuals, except for thoracic extension, which was greater in children with achondroplasia. Segmental spinal postures were different between children with achondroplasia and healthy children, whereas no differences between the two groups were observed in global spinal postures, implying the importance of considering segmental spinal postures in the assessment of achondroplasia in childhood. These findings demonstrate that spinal mobility appears to be more influenced by achondroplasia than global spinal postures in childhood. These results also highlight the importance of considering the musculoskeletal assessment of segmental spinal postures and rehabilitative interventions aimed at promoting spinal and hip flexibility in children with achondroplasia.

## Figures and Tables

**Table 1:** Demographic and anthropometric characteristics of the study groups (mean ± standard deviations).

Variables	Children with Achondroplasia (N = 13)	Healthy Children (N = 13)	*p*-Value
Age (years)	10.6 (2.8)	11.1 (2.6)	0.57 ^w^
Sex (female)	38%	38%	1 ^c^
Weight (kg)	25.9 (9.6)	41.3 (14.0)	0.003 ^I^
Height (cm)	110.1 (18.1)	146.3 (15.3)	<0.0001 ^I^
BMI (kg/m^2^)	20.7 (2.6)	18.6 (2.7)	0.06 ^I^
Spinal length (mm)	353.1 (46.4)	387.7 (41.0)	0.56 ^I^

*p*-value—the significance of differences between the two groups was calculated by the (^I^) independent samples *t*-test, (^w^) Wilcoxon’s rank sum test and the (^c^) Pearson’s chi-square test. BMI—body mass index.

**Table 2:** Differences in spinal postures, lumbar, thoracic, and hip mobility between children with achondroplasia and age- and spinal length-matched healthy children.

Variables	Children with Achondroplasia n = 13		Healthy Children n = 13		Differences in Spinal Posture and Mobility (95% CI)	*p*-Value
	EMM	SE	EMM	SE		
Spinal postures						
Thoracic Kyphosis (Th1-12)	25.1	3.9	31.2	3.9	−6.1 (−17.6 to 5.3)	0.27
Lumbar lordosis	23.4	3.8	27.7	3.8	−4.3 (−6.8 to 15.5)	0.43
Sacral kyphosis	15.8	3.4	17.9	3.4	−2.1 (−12.1 to 7.9)	0.66
Posture of each individual spinal segment						
T1/2	3.9	0.6	2.2	0.6	1.6 (−0.6 to 3.6)	0.08
T2/3	3.3	1.1	3.3	1.1	0.001 (−3.1 to 3.1)	0.99
T3/4	2.8	0.6	4.4	0.6	−1.6 (−3.5 to 0.2)	0.08
T4/5	3.4	0.5	4.3	0.5	−0.8 (−2.4 to 0.7)	0.26
T5/6	1.8	0.6	2.8	0.6	−1.1 (−2.8 to 0.7)	0.22
T6/7	0.6	0.6	3.2	0.6	−2.6 (−4.6 to −0.6)	0.01 *
T7/8	0.8	0.8	3.7	0.8	−2.9 (−5.2 to −0.6)	0.02 *
T8/9	2.2	0.5	4.0	0.5	−1.8 (−3.4 to −0.2)	0.02 *
T9/10	1.9	0.5	3.1	0.5	−1.2 (−2.6 to 0.2)	0.08
T10/11	1.3	0.8	1.7	0.8	−0.4 (−2.7 to 1.8)	0.71
T11/12	2.7	0.8	−0.5	0.8	2.7 (0.4 to 4.9)	0.02 *
T12/L1	1.2	0.6	−0.7	0.6	2.0 (0.1 to 3.9)	0.03 *
L1/2	−0.5	1.1	−1.9	1.1	1.4 (−1.8 to 4.7)	0.36
L2/3	−2.9	1.1	−3.4	1.1	0.5 (−2.7 to 3.6)	0.75
L3/4	−4.8	1.0	−7.3	1.0	2.4 (−0.5 to 5.4)	0.11
L4/5	−7.2	0.9	−8.4	0.9	1.3 (−1.5 to 4.0)	0.35
L5/S1	−8.4	1.4	−7.1	1.4	−1.3 (−5.3 to 2.8)	0.52
Spinal mobility						
Thoracic (°)						
Flexion	24.3	4.2	25.6	4.2	−1.3 (−13.6 to 11.0)	0.83
Extension	19.4	4.2	2.9	4.2	16.5 (4.4 to 28.7)	0.009 *
Lateral flexion	41.1	6.9	61.4	6.9	−20.4 (−40.6 to −0.1)	0.04 *
Lumbar (°)						
Flexion	47.0	4.1	64.4	4.1	−17.4 (−29.4 to −5.4)	0.006 *
Extension	1.7	2.9	15.9	2.9	−14.2 (−22.8 to −5.7)	0.002 *
Lateral flexion	17.3	3.0	36.9	3.0	−19.6 (−28.4 to −10.7)	0.0001 *
Hip mobility						
Hip (°)						
Flexion	43.7	5.8	41.7	5.8	2.0 (−14.9 to 18.9)	0.81
Extension	5.2	3.3	17.1	3.3	−11.9 (−2.4 to −21.5)	0.01 *
Lumbar-to-hip ratio	0.5	0.04	0.6	0.04	−0.08 (−0.22 to 0.05)	0.22

*p*-value (adjusted for age and sex)—the significance of differences between the two groups. (*) indicates a p value of less than 0.05. EMM estimated marginal means for the sum of the respective range of motion values in each spinal segment in the different spinal regions. SE standard errors. CI confidence interval. The lumbar-to-hip ratio was calculated by dividing the lumbar range of motion by the sum of the lumbar range of motion and the hip range of motion during the trunk flexion in the sagittal plane. “T” and “L” indicate thoracic and lumbar vertebrae, respectively. (“/”) is placed between two adjacent vertebrae creating segmental postures. For example, T1/2 indicates the segmental angle formed by the first and second thoracic vertebrae.

## Data Availability

Raw data will be available on www.zenodo.org after the acceptance of the manuscript upon a reasonable request to the corresponding author.
